# Hematopoietic Growth Factors Regulate the Entry of Monocytes into the Adult Brain via Chemokine Receptor CCR5

**DOI:** 10.3390/ijms25168898

**Published:** 2024-08-15

**Authors:** Xuefang Sophie Ren, Junchi He, Songruo Li, Heng Hu, Michele Kyle, Shinichi Kohsaka, Li-Ru Zhao

**Affiliations:** 1 Department of Neurology, Cellular Biology and Anatomy, Louisiana State University Health Sciences Center, Shreveport, LA 71130, USA; 2 Department of Neurosurgery, The State University of New York Upstate Medical University, Syracuse, NY 13210, USA; 3 National Institute of Neuroscience, 4-1-1 Ogawahigashi, Kodaira, Tokyo 187-8502, Japan

**Keywords:** endothelial cells, monocytes, macrophages, hematopoietic growth factors, adhesion, stem cell factor, granulocyte colony-stimulating factor, chemokine receptor 5

## Abstract

Monocytes are circulating macrophage precursors generated from bone marrow hematopoietic stem cells. In adults, monocytes continuously replenish cerebral border-associated macrophages under physiological conditions. Monocytes also rapidly infiltrate the brain in pathological settings. The mechanisms of recruiting monocyte-derived macrophages into the brain under pathological conditions have been extensively studied. However, it remains unclear how monocytes enter the brain to renew border-associated macrophages under physiological conditions. Using both in vitro and in vivo approaches, this study reveals that a combination of two hematopoietic growth factors, stem cell factor (SCF) and granulocyte colony-stimulating factor (G-CSF), complementarily and synergistically enhances the adhesion of monocytes to cerebral endothelial cells in a dose-dependent manner. Cysteine-cysteine chemokine receptor 5 (CCR5) in brain endothelial cells, but not the cell adhesion molecules mediating neuroinflammation-related infiltration of monocyte-derived macrophages, modulates SCF+G-CSF-enhanced monocyte-endothelial cell adhesion. Blocking CCR5 or genetically deleting CCR5 reduces monocyte-endothelial cell adhesion induced by SCF+G-CSF. The SCF+G-CSF-enhanced recruitment of bone marrow-derived monocytes/macrophages into the cerebral perivascular space is also reduced in adult CCR5 knockout mice. This study demonstrates the role of SCF and G-CSF in regulating the entry of monocytes into the adult brain to replenish perivascular macrophages.

## 1. Introduction

The vascular endothelium, a monolayer of endothelial cells, forms the inner cellular wall of blood vessels and mediates many diverse biological processes including the maintenance of an anti-thrombotic surface between the circulating blood and tissue [1], the regulation of vascular tone through the release of vasodilator and vasoconstrictive agents [2,3], and the selective separation of vascular luminal contents from tissues through barrier formation [4]. The blood-brain barrier, formed by endothelial cells, plays a vital role in maintaining the homeostasis of the brain microenvironment [5].

Macrophages in the brain consist of microglia in the parenchyma, border-associated macrophages, and monocyte-derived macrophages that enter the brain under pathological conditions [6]. Both microglia and border-associated macrophages in the meningeal-choroid plexus-perivascular space are of embryonic origin, while the border-associated macrophages are renewed through monocyte recruitment under physiological conditions in the adult brain [6]. The mechanisms underlying the recruitment of monocyte-derived macrophages into the brain under pathological conditions have been extensively studied [5,6]. However, it remains poorly understood how monocytes enter the adult brain to renew the border-associated macrophages under physiological conditions.

Stem cell factor (SCF, also termed master cell growth factor, kit ligand, and steel factor) and granulocyte colony-stimulating factor (G-CSF) are produced mainly by bone marrow stromal cells [7,8]. SCF and G-CSF are essential hematopoietic growth factors. SCF, in combination with, G-CSF shows synergistic effects in enhancing the proliferation, differentiation, survival, and mobilization of hematopoietic stem cells [9,10,11]. SCF binds to its specific receptor, Ckit, to regulate various biological functions such as hematopoiesis, gametogenesis, and melanogenesis [12,13,14,15,16]. G-CSF interacts with its receptor, GCSFR, to control the proliferation, differentiation, and maturation of the precursor cells of granulocytes [17,18,19]. However, increasing evidence has revealed that SCF and G-CSF also have biological functions in the central nervous system. It has been shown that combined treatment with SCF and G-CSF enhances neurogenesis and cerebral angiogenesis [20,21,22,23] and synergistically increases neurite outgrowth [24]. The expression of SCF and G-CSF receptors has been found on cerebral endothelial cells [25]. However, the exact biological function of SCF and G-CSF on cerebral endothelial cells has not been fully understood. 

Monocytes, expressing specific markers such as ionized calcium-binding adaptor molecule 1 (Iba-1), CD115, and CD11b [26,27], are a type of white blood cell that plays multiple roles in the immune system, including replenishing resident macrophages and dendritic cells under normal or pathological conditions [28,29]. It is thought that monocyte recruitment into tissue may follow the general processes of adhesion and trafficking, which include rolling, adhesion, and transmigration across vascular endothelial cells [30,31]. The adhesion molecules expressed on vascular endothelial cells are crucially involved in leukocyte infiltration during neuroinflammation in various disease conditions [5,32]. However, it remains unclearwhether SCF and G-CSF could promote the expression of adhesion molecules or other molecules on cerebral vascular endothelial cells to recruit circulating monocytes into the brain and replace the border-associated macrophages under physiological conditions. Using both in vitro and in vivo approaches, the purpose of this study is to determine the effects and mechanisms of SCF and G-CSF in regulating monocyte-endothelial cell adhesion and the entry of monocytes into the adult brain under physiological conditions.

## 2. Results

### 2.1. Receptors for SCF and G-CSF Are Expressed on Brain Endothelial Cells

**Figure 1:**
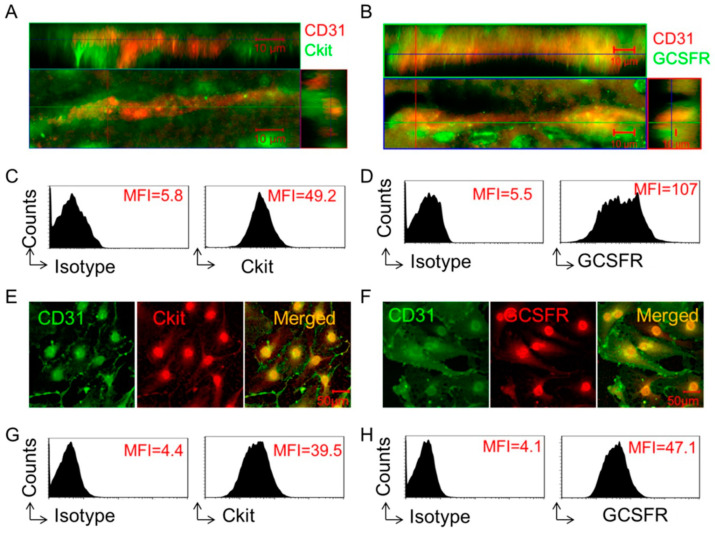
Receptors for SCF and G-CSF are expressed on brain endothelial cells. (**A**,**B**) Representative 3-dimensional (3D) confocal images. Capillaries (CD31^+^, red) in the cortex of the adult mouse brain show co-expression with the receptors for SCF (**A**) (Ckit, green) and G-CSF (**B**) (GCSFR, green). Scale bar, 10 µm. (**C**,**D**) Representative flow cytometry data. The expressions of Ckit (**C**) and GCSFR (**D**) on cerebral endothelial cells are detected by flow cytometry analysis of CD45^−^CD31^+^ gated endothelial cells isolated from adult mouse brain. Mean fluorescence intensity (MFI) of Ckit (**C**) and GCSFR (**D**) expressing on cerebral endothelial cells is presented in flow cytometry histograms. Isotype: isotype control antibody. Repeated experiments, n = 4. (**E**,**F**) Representative confocal images of immunofluorescence staining. The expression of Ckit (**E**) (red) and GCSFR (**F**) (red) are seen on bEnd.3 endothelial cells (CD31^+^, green). Scale bar, 50 µm. Repeated experiments, n = 3. (**G**,**H**) Representative flow cytometry histograms showing the expression of Ckit (**G**) and GCSFR (**H**) on bEnd.3 endothelial cells. MFI: Mean fluorescence intensity. Isotype: isotype control antibody. Repeated experiments, n = 5.

To determine and validate the expression of receptors for SCF and G-CSF on brain endothelial cells, we performed immunofluorescence staining and flow cytometric analysis on adult mouse brain tissue and mouse brain-derived endothelial cells. Immunofluorescence staining of brain sections revealed that the SCF receptor (Ckit) and G-CSF receptor (GCSFR) were co-localized with CD31^+^ endothelial cells in the cortex (Figure 1A,B). Flow cytometry analysis showed the mean fluorescence intensity (MFI) of Ckit as 49.2 and GCSFR as 107 on CD45^−^CD31^+^ gated primary endothelial cells isolated from the adult mouse brain (Figure 1C,D). In addition, we found that both Ckit and GCSFR were expressed on bEnd.3 cells (an endothelial cell line derived from the mouse brain), which was confirmed by both immunofluorescence staining (Figure 1E,F) and flow cytometry (Figure 1G,H). These validated data demonstrate that the receptors for SCF and G-CSF are constitutively expressed on endothelial cells, suggesting that SCF and G-CSF might have important biological functions in these cells.

### 2.2. SCF in Combination with G-CSF Enhances Monocyte Adhesion to Endothelial Cells In Vitro

As stated earlier, under physiological conditions, monocytes contribute to the generation of macrophages in the adult brain [6,33,34]. However, it is not known how monocytes enter the adult brain. To address this question, we determined the effects of SCF and G-CSF on the regulation of monocyte adhesion to endothelial cells. 

**Figure 2:**
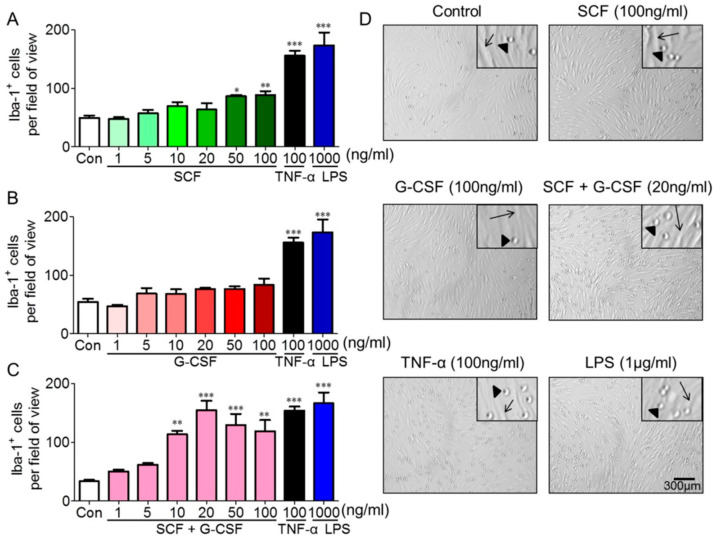
SCF in combination with G-CSF enhances Iba-1^+^ monocyte adhesion to endothelial cells. (**A**–**C**) Monocyte-endothelial cell adhesion data showing Iba-1^+^ monocytes adhering to bEnd.3 endothelial cells pre-treated with different doses of SCF alone (**A**), G-CSF alone (**B**), or SCF+G-CSF (**C**) for 16–18 h. Inflammatory mediators TNF-α and LPS serve as positive controls. Repeated by three independent experiments. Each experiment includes three samples (cell culture wells). Mean ± SEM. * *p* < 0.05, ** *p* < 0.01, *** *p* < 0.001, vs. medium controls. One-way ANOVA followed by post hoc Tukey’s test. (**D**) Representative bright-field images showing the adhesion of Iba-1^+^ monocytes on bEnd.3 endothelial cells pre-treated with medium (control), SCF (100 ng/mL), G-CSF (100 ng/mL), SCF+G-CSF (20 ng/mL), TNF-α (100 ng/mL), and LPS (1 µg/mL). Arrows indicate reference points illustrating spindle-shaped endothelial cells. Triangles denote round Iba-1^+^ monocytes. Scale bar, 300 µm.

Initially, we examined whether SCF alone promotes the adhesion of monocytes to endothelial cells. For this purpose, we conducted adhesion experiments using bEnd.3 cells and monocytes. Brain-derived endothelial cells (bEnd.3 cells) were pre-treated with SCF at different dosages for 16–18 h, after which bone marrow-derived monocytes were loaded onto the pre-treated bEnd.3 cells. The bone marrow-derived monocytes (Iba-1^+^ cells) were isolated from the bone marrow of transgenic mice carrying Iba1-enhanced green fluorescent protein (GFP) (Iba1-GFP mice). After a 20-min incubation, an adhesion assay was performed. We observed that significantly greater amounts of monocytes adhered to the endothelial cells pre-stimulated with SCF at 50 ng/mL (*p* < 0.05) and 100 ng/mL (*p* < 0.01) compared to endothelial cells pre-treated with medium alone or a lower concentration of SCF (Figure 2A,D). These findings reveal that SCF enhances monocyte adhesion to endothelial cells.

Next, we determined whether G-CSF alone has similar effects on enhancing endothelial cell/monocyte adhesion. Surprisingly, G-CSF alone did not show significant changes in the adhesion of Iba-1^+^ monocytes to endothelial cells at any tested dosage (Figure 2B,D). In contrast, in the positive controls, significantly increased numbers of monocytes adhering to bEnd.3 cells were observed following a 16-h pre-treatment with 100 ng/mL TNF-α (*p* < 0.001) or 1 µg/mL LPS (*p* < 0.001) compared to the medium controls (Figure 2). These experiments were repeated four times with similar results.

Furthermore, we evaluated the efficacy of a combination of SCF and G-CSF (SCF+G-CSF) on endothelial cell/monocyte adhesion. Remarkably, SCF+G-CSF strongly enhanced Iba-1^+^ monocyte adhesion to bEnd.3 endothelial monolayers compared to medium controls (Figure 2C,D). The binding of monocytes to bEnd.3 endothelial cells peaked at a dose of 20 ng/mL SCF and 20 ng/mL G-CSF (*p* < 0.001). Although significant increases in monocyte/endothelial cell adhesion were still observed at SCF and G-CSF doses of 50 ng/mL (*p* < 0.001) and 100 ng/mL (*p* < 0.01), the levels of adhesion slightly decreased as the SCF+G-CSF dose increased (Figure 2C). These findings indicate that SCF+G-CSF enhances monocyte adhesion to endothelial cells in a dose-dependent manner. Notably, SCF or G-CSF alone at a dose of 20 ng/mL did not increase the adhesion of Iba-1^+^ monocytes to endothelial cells (Figure 2A,B). To validate these findings, we used flow cytometry to examine the differences between the adhesion of Iba-1-GFP^+^ monocytes to untreated/unstimulated bEnd.3 cells (control) and bEnd.3 cells pre-treated/pre-stimulated with SCF+G-CSF (20 ng/mL), TNF-α (100 ng/mL), or LPS (1 µg/mL). As shown in Supplementary Figure S1, Iba-1-GFP^+^ cells binding to SCF+G-CSF-pre-treated/pre-stimulated bEnd.3 cells were significantly increased compared to untreated/unstimulated bEnd.3 cells (*p* < 0.01). Iba-1-GFP^+^ cells showed significantly higher adhesion ratios to TNF-α or LPS pre-stimulated bEnd.3 cells than to untreated/unstimulated bEnd.3 cells (*p* < 0.001). These data strongly suggest that SCF in combination with G-CSF has a synergistic effect in promoting the adhesion function of endothelial cells. 

### 2.3. SCF+G-CSF Upregulates the Expression of CCR5 but Not Adhesion Molecules in Endothelial Cells

**Figure 3:**
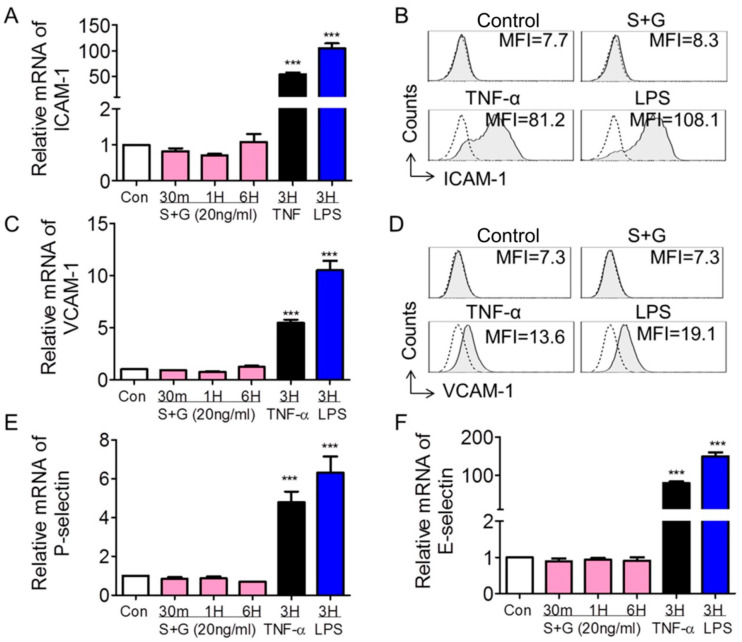
Expression of adhesion molecules in bEnd.3 endothelial cells. (**A**,**C**,**E**,**F**) Quantitative real-time PCR data showing the mRNA expression of ICAM-1 (**A**), VCAM-1 (**C**), P-selectin (**E**), and E-selectin (**F**) in bEnd.3 endothelial cells cultured for different periods (30 min to 6 h) in the presence of medium alone (con: control), SCF+G-CSF (20 ng/mL), TNF-α (100 ng/mL), and LPS (1 µg/mL). Repeated by three independent experiments. Mean ± SEM. *** *p* < 0.001 vs. medium control. One-way ANOVA followed by post hoc Tukey’s test. (**B**,**D**) Representative flow cytometry histograms showing membrane expression levels of ICAM-1 (**B**) and VCAM-1 (**D**) on bEnd.3 cells with different treatments. MFI: Mean fluorescence intensity. Repeated experiments, n = 5. Dashed lines indicate isotype IgG controls. Filled gray lines represent specific staining for the indicated adhesion molecules.

Under inflammatory conditions, many adhesion molecules are expressed in endothelial cells; these adhesion molecules play a critical role in the rolling, adhesion, and migration of leukocytes across the vascular endothelial barrier [32,35]. Here, we used real-time qPCR and flow cytometry to determine whether SCF+G-CSF increases the expression of adhesion molecules in endothelial cells. We observed that the gene expression levels of vascular cell adhesion molecule-1 (VCAM-1, CD106), intracellular adhesion molecule-1 (ICAM-1, CD54), P-selectin, and E-selectin were markedly upregulated in bEnd.3 cells after 3 h treatment with the inflammatory mediators TNF-α and LPS (*p* < 0.001) (Figure 3). However, SCF+G-CSF treatment did not significantly change the gene expression levels of ICAM-1, VCAM-1, P-selectin, and E-selectin in bEnd.3 cells (Figure 3). Flow cytometric analysis of ICAM-1 and VCAM-1 expression on bEnd.3 cells was consistent with the results from real-time qPCR (Figure 3B,D). These findings suggest that the SCF+G-CSF-enhanced adhesion function of vascular endothelial cells is mediated through a pathway distinct from inflammatory signaling.

**Figure 4:**
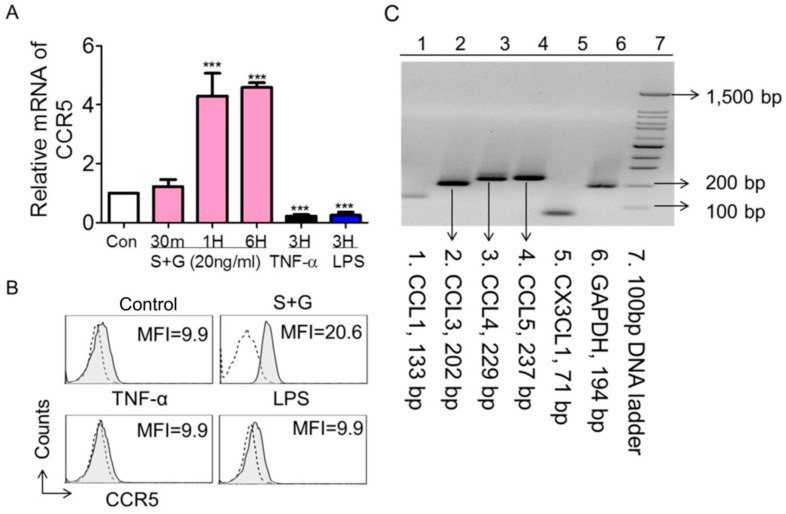
SCF+G-CSF increases CCR5 expression on bEnd.3 endothelial cells. (**A**) Quantitative real-time PCR data showing mRNA expression of CCR5 in bEnd.3 endothelial cells cultured for different periods (30 min to 6 h) in the presence of medium alone (con: control), SCF+G-CSF (20 ng/mL), TNF-α (100 ng/mL), and LPS (1 µg/mL). Mean ± SEM. *** *p* < 0.001 vs. medium control. One-way ANOVA followed by post hoc Tukey’s test. (**B**) Representative flow cytometry histograms showing membrane expression of CCR5 on bEnd.3 endothelial cells. MFI: Mean fluorescence intensity. Repeated experiments, n = 5. Dashed lines indicate isotype IgG controls. Filled gray lines represent specific staining for CCR5. (**C**) PCR-electrophoresis showing mRNA expression of CCL1 (133 bp), CCL3 (202 bp), CCL4 (229 bp), CCL5 (237 bp), and CX3CL1 (71 bp) in Iba-1^+^ monocytes.

Chemokines and chemokine receptors are structurally related proteins that regulate leukocyte adhesion and trans-endothelial migration both in vitro and in vivo [36,37]. To further investigate the possible mechanisms underlying SCF+G-CSF-enhanced monocyte/endothelial cell adhesion, we examined the alterations in chemokine receptors on bEnd.3 cells following SCF+G-CSF treatment using real-time qPCR. Our data showed that the mRNA levels of chemokine receptors CCR1, CCR2, CCR3, CCR4, CCR8, and CXCR4 were very low in bEnd.3 cells, and 20 ng/mL SCF+G-CSF treatment did not increase the mRNA expression of these chemokine receptors (Supplementary Figure S2), with the exception of CCR5. When bEnd.3 cells were treated with 20 ng/mL SCF+G-CSF, the mRNA expression of CCR5 was significantly increased compared to medium controls (*p* < 0.001) (Figure 4A). This finding was further validated by flow cytometry. SCF+G-CSF treatment increased CCR5 expression on bEnd.3 cells (Figure 4B). Interestingly, the inflammatory mediators TNF-α and LPS significantly reduced CCR5 mRNA expression in bEnd.3 cells compared to medium controls (*p* < 0.001) (Figure 4A). 

### 2.4. SCF+G-CSF Enhances Monocyte Adhesion to Endothelial Cells through CCR5 In Vitro

CCR5 binds to several chemokine ligands, including CCL3, CCL4, and CCL5 [38,39,40]. In studying cell-cell adhesion, it is important to detect whether Iba-1^+^ monocytes express the ligands of CCR5 when CCR5 receptors are increased on endothelial cells by SCF+G-CSF. Our data revealed that Iba-1^+^ monocytes constitutively express CCL3, CCL4, and CCL5 (Figure 4C). These data suggest that the CCR5-related chemokine-receptor pathway could be an important mechanism underlying the adhesion of monocytes to SCF+G-CSF-treated endothelial cells.

**Figure 5:**
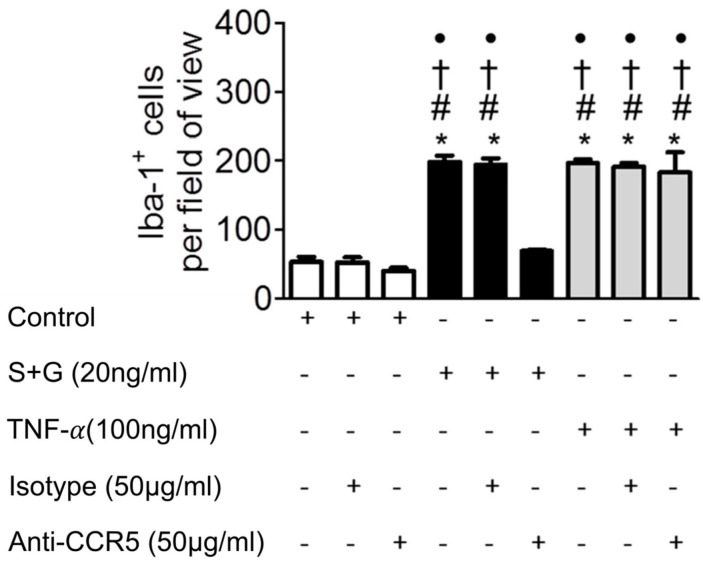
Anti-CCR5 antibody blocks SCF+G-CSF-enhanced monocyte adhesion to endothelial cells. Monolayers of mouse brain-derived endothelial cells (i.e., bEnd.3 cells) were incubated in the presence of medium alone (control), SCF+G-CSF (20 ng/mL), and TNF-α (100 ng/mL) for 16–18 h. After washing, the bEnd.3 cells were pre-treated with anti-CCR5 antibody or isotype IgG control antibody before adding Iba-1^+^ monocytes to the bEnd.3 cell monolayers. Mean ± SEM. N = 7. * *p* < 0.001 vs. medium control, # *p* < 0.001 vs. medium control with isotype control, † *p* < 0.001 vs. medium control with anti-CCR5, and • *p* < 0.001 vs. S+G with anti-CCR5. One-way ANOVA followed by post hoc Tukey’s test.

We then sought to determine the role of endothelial CCR5 in SCF+G-CSF-enhanced monocyte-endothelial adhesion. To block CCR5, after pre-treatment of bEnd.3 monolayers with SCF+G-CSF (20 µg/mL), anti-CCR5 antibody (50 µg/mL) or its isotype IgG2a control (50 µg/mL) was added to the washed bEnd.3 cells. After washing out the antibodies with pre-warmed medium, FACS-sorted Iba-1-GFP^+^ monocytes were added to the bEnd.3 monolayers. Remarkably, the SCF+G-CSF-increased monocyte/endothelial cell adhesion was completely blocked by anti-CCR5. The control antibody against IgG2a did not affect the adhesion of monocytes to SCF+G-CSF-treated bEnd.3 cells (Figure 5). The anti-CCR5 antibody did not block the adhesion of Iba-1^+^ monocytes to the inflammatory cytokine TNF-α-stimulated bEnd.3 cells. These data strongly suggest that endothelial CCR5 controls the adhesion of monocytes to SCF+G-CSF-pre-treated endothelial cells, which is entirely different from an inflammatory signaling-mediated pathway for monocyte-endothelial cell adhesion.

### 2.5. SCF+G-CSF Enhances Bone Marrow-Derived Cells Adhering to Cerebral Endothelial Cells through CCR5 In Vivo

**Figure 6:**
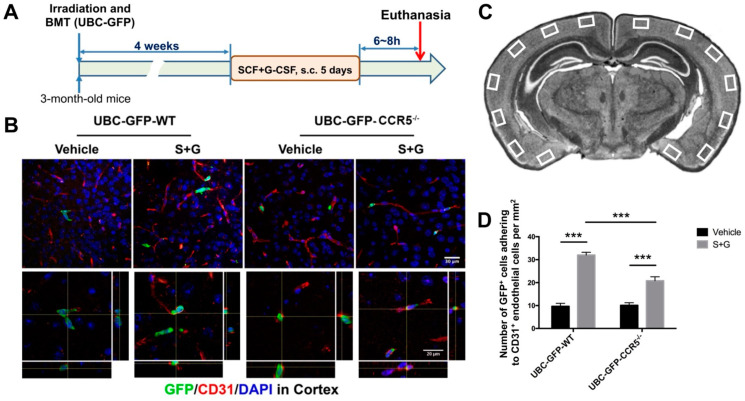
SCF+G-CSF increases adhesion of bone marrow-derived cells to cerebral endothelial cells partially through CCR5 in adult mice. (**A**) Experimental flowchart. BMT: bone marrow transplantation. Bone marrow donor: UBC-GFP mice. (**B**) Representative confocal images showing immunofluorescence staining of GFP-positive bone marrow-derived cells (green) and CD31-positive endothelial cells (red) in the cortex of UBC-GFP bone marrow-transplanted vehicle-control WT mice, vehicle-control CCR5^−/−^ mice, SCF+G-CSF-treated WT mice, and SCF+G-CSF-treated CCR5^−/−^ mice. Z-stack images of 15 layers with 1 μm intervals. DAPI (blue): nuclear counterstaining. Upper 4 images: Projection views of z-stack images (Scale bar, 30 µm). Lower 4 images: Orthogonal views of z-stack images showing 3D visualizations of the colocalization of bone marrow-derived cells (GFP, green) with endothelial cells (CD31, red). Scale bar, 20 µm. (**C**) A diagram showing the selected regions in the cortex for confocal imaging. (**D**) Quantification data showing the number of GFP^+^ cells adhering to CD31^+^ endothelial cells per mm^2^ in the experimental groups. Mean ± SEM. N = 5, *** *p* < 0.001. One-way ANOVA followed by post hoc Tukey’s test.

To verify that SCF+G-CSF treatment increases blood cell adhesion to endothelial cells under physiological conditions in vivo, we created UBC-GFP^+^ bone marrow chimeric CCR5^−/−^ and wild-type (WT) mice (i.e., UBC-GFP-CCR5^−/−^ mice and UBC-GFP-WT mice). In these mice, we examined the adhesion of bone marrow-derived cells (GFP-positive) to cerebral endothelial cells (CD31-positive) after SCF+G-CSF treatment (experimental flow chart, Figure 6A). We observed that SCF+G-CSF treatment increased bone marrow-derived GFP^+^ cell adhesion to CD31^+^ endothelial cells in the cerebral cortex of both UBC-GFP-WT mice and UBC-GFP-CCR5^−/−^ mice compared to the vehicle control groups (*p* < 0.001). However, the number of GFP^+^ cells adhering to endothelial cells was significantly lower in SCF+G-CSF-treated UBC-GFP-CCR5^−/−^ mice than in SCF+G-CSF treated UBC-GFP-WT mice (*p* < 0.001) (Figure 6). These findings suggest that SCF+G-CSF-enhanced adhesion of bone marrow-derived blood cells to brain endothelial cells is, at least partially, mediated through CCR5.

### 2.6. CCR5 Mediates Bone Marrow-Derived Monocyte Transmigration into the Brain Surrounding Blood Vessels

**Figure 7:**
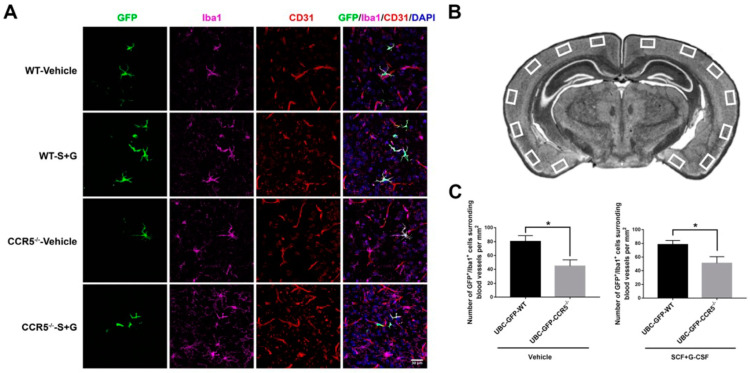
CCR5 mediates bone marrow-derived monocyte transmigration into the adult brain. (**A**) Representative projection views of z-stack confocal images showing the location of bone marrow-derived monocytes (GFP^+^/Iba1^+^ cells) next to the blood vessels (CD31^+^) in the cerebral cortex of adult mice that received UBC-GFP bone marrow transplantation in the groups of vehicle-control WT mice, vehicle-control CCR5^−/−^ mice, SCF+G-CSF-treated WT mice, and SCF+G-CSF-treated CCR5^−/−^ mice. DAPI (blue): nuclear counterstaining. Scale bar, 30 µm. Z-stack images of 15 layers with 1 μm intervals. (**B**) A diagram showing the selected regions in the cortex for confocal imaging. (**C**) Quantification data. The number of GFP^+^/Iba1^+^ bone marrow-derived monocytes/macrophages surrounding CD31^+^ blood vessels (within 10 μm from the blood vessels) in the cerebral cortex is reduced in both vehicle-control CCR5^−/−^ mice and SCF+G-CSF-treated CCR5^−/−^ mice. Mean ± SEM. N = 5. * *p* < 0.05, Unpaired *t*-test.

To identify whether CCR5 modulates bone marrow-derived monocyte transmigration into the brain via SCF+G-CSF treatment, we quantified the number of GFP^+^/Iba1^+^ cells surrounding the CD31^+^ endothelial cells in the cortex (surrounding: within 10 μm from vessels). In the vehicle control mice, the number of GFP^+^/Iba1^+^ cells surrounding the blood vessels in the cerebral cortex was significantly reduced in CCR5 knockout mice compared to WT mice (*p* < 0.05) (Figure 7). Similarly, in the SCF+G-CSF-treated mice, the number of GFP^+^/Iba1^+^ cells surrounding the blood vessels in the cortex was significantly decreased in CCR5 knockout mice compared to WT mice (*p* < 0.05) (Figure 7). These data suggest that CCR5 is involved in the transmigration of Iba1^+^ bone marrow-derived monocytes into the brain and that CCR5 is required for SCF+G-CSF-enhanced transmigration of blood monocytes into the brain. Interestingly, GFP^+^/Iba1^+^ cells were found only around, but not inside, the lumen of CD31^+^ brain vessels, indicating that monocytes may differentiate into Iba1^+^ macrophages immediately after transmigrating from the blood vessels into the brain.

To further determine whether CCR5 and/or SCF+G-CSF treatment affects the proliferation of infiltrated bone marrow-derived monocytes/macrophages (GFP^+^/Iba1^+^ cells), we performed triple immunofluorescence staining to detect Ki67/Iba1/GFP-positive cells. Our findings showed that neither CCR5 nor SCF+G-CSF treatment changes the number of Ki67/Iba1/GFP-positive cells (Supplementary Figure S3).

## 3. Discussion

In the present study, we have, for the first time, identified that the combination of two hematopoietic growth factors, SCF and G-CSF (i.e., SCF+G-CSF), can enhance the replenishment of perivascular macrophages in the adult mouse brain via CCR5 under physiological conditions. The findings of our in vitro experiments reveal that SCF+G-CSF complementarily and synergistically promotes the adhesion function of brain endothelial cells to bind with bone marrow-derived monocytes. The SCF+G-CSF-enhanced adhesion function of brain endothelial cells is mediated through CCR5, not through cell adhesion molecules. Our findings demonstrate that SCF+G-CSF-enhanced and CCR5-mediated monocyte-endothelial cell adhesion is distinct from the monocyte-endothelial cell adhesion stimulated by an inflammatory signaling-triggered *cell adhesion molecule*-dependent pathway. The findings of our in vivo experiments further confirm the role of CCR5 in modulating SCF+G-CSF-enhanced renewal of perivascular macrophages in the adult brain by bone marrow-derived monocytes under physiological conditions.

SCF and G-CSF act on their target cells through specific membrane receptors to regulate many cell functions, including promoting neurite outgrowth [24,41] and inhibiting apoptosis [42]. In the present study, we found that the receptors for SCF and G-CSF are expressed in endothelial cells of the adult mouse brain and in the brain-derived endothelial cell line. This observation is consistent with a previous report showing that the SCF receptor, Ckit, and the G-CSF receptor, GCSFR, are expressed in cerebral endothelial cells [25]. These findings implicate the ability of SCF and G-CSF to modulate endothelial cell functions.

Using a well-established cell adhesion model in vitro, our novel finding reveals that SCF+G-CSF exhibits a synergistic effect on monocyte-endothelial cell adhesion greater than either of them alone. The optimal dose for increasing monocyte-endothelial cell adhesion peaked at 20 ng/mL of SCF+G-CSF in vitro, and the number of adherent monocytes was not further increased by increasing the dose. Notably, SCF in combination with G-CSF also shows synergistic effects in enhancing the proliferation, differentiation, survival, and mobilization of hematopoietic stem cells [9,10,11] and in promoting neurite outgrowth [24]. The mechanism underlying the synergistic effects of SCF+G-CSF in the regulation of cell functions is not clear. One possibility is that the proper doses of SCF+G-CSF that increase monocyte-endothelial cell binding may robustly activate their receptor-mediated cell signaling because either SCF-Ckit or G-CSF-GCSFR interaction leads to multiple signaling cascades, including the RAS/ERK, PI3-K/AKT, Src Kinase, JAK/STAT, and MEK/ERK pathways [24,43,44,45]. Both SCF and G-CSF share some similar signaling pathways but each of them could have relatively independent signaling pathways. Our data show that G-CSF alone does not affect the adhesion of monocytes to endothelial cells when the endothelial cells lack the necessary signaling triggered by SCF. The precise mechanism by which this occurs remains unclear. It has been shown that SCF+G-CSF synergistically increases myeloid cell proliferation through complementary signaling pathways [9]. G-CSF, but not SCF, induces the tyrosine phosphorylation of STAT1 and STAT3 signaling. However, SCF induces the phosphorylation of STAT3 on serlne727, which is required for maximal and complete STAT3 transcriptional activity when SCF is combined with G-CSF [9]. The underlying molecular mechanisms of the SCF+G-CSF-induced synergistic effects in enhancing endothelial cell adhesion function when binding with monocytes, however, need further investigation.

It is widely known that inflammatory signals initiate the expression of adhesion molecules on the endothelial cell surface (such as ICAMs, VCAMs, P-selectin and E-selectin) [32,36,46]. These adhesion molecules are responsible for leukocyte-endothelial cell crosstalk and mediate the entry of leukocytes into tissues under pathological conditions [47,48,49,50,51]. While the initial phase of leukocyte-endothelial cell adhesion is not entirely clear, leukocyte recruitment is undoubtedly of fundamental importance and remains a predominant feature during inflammation [52]. Remarkably, our study demonstrates that SCF+G-CSF fails to induce changes in inflammation-related cell adhesion molecules, such as ICAM, VCAM, P-selectin, or E-selectin, despite leading to robust increases in monocyte-endothelial cell adhesion. These findings suggest that the molecules mediating the adhesion of monocytes to endothelial cells via SCF+G-CSF are entirely different from those involved in inflammation-induced cell adhesion. Our in vivo experiment confirms that SCF+G-CSF treatment increases cerebral endothelial cell adhesion to bone marrow-derived cells in healthy adult wild-type mice, supporting the role of SCF+G-CSF in enhancing monocyte-endothelial cell adhesion under physiological conditions.

It has been demonstrated that chemokines and chemokine receptors regulate leukocyte adhesion and trans-endothelium migration both in vitro and in vivo [53,54,55]. Activation signals from chemokines and chemokine receptors initiate the firm adhesion of leukocytes to endothelial cells by rapidly upregulating integrin affinity [56,57]. In the present study, we screened chemokine receptors expressed by endothelial cells under SCF+G-CSF stimulation. Our data show that CCR5 is pivotal in controlling the monocyte-endothelial cell adhesion induced by SCF+G-CSF. It has been shown that CCR5 is expressed in different types of cells, including endothelial cells [58]. The natural chemokine ligands that interact with CCR5 include CCL5 (also known as RANTES), macrophage inflammatory protein (MIP)-1α, MIP-1β (also known as CCL3 and CCL4), and CCL3L1 [38,39,40,59]. In the present study, these CCR5 chemokine ligands are detectable on bone marrow-derived Iba-1^+^ monocytes, suggesting the possibility of monocyte-endothelial cell interaction via CCR5-related ligand-receptor binding. Our data also reveal that the expression of CCR5 on endothelial cells is upregulated by SCF+G-CSF treatment in vitro. Blocking CCR5 on endothelial cells eliminates the SCF+G-CSF-induced binding of monocytes to endothelial cells, demonstrating that CCR5 plays an important role in SCF+G-CSF-enhanced adhesion of monocytes to endothelial cells. These findings are further validated by our in vivo study. CCR5 knockout mice that received bone marrow transplantation from UBC-GFP mice exhibited decreased adhesion of bone marrow-derived GFP^+^ cells to endothelial cells induced by SCF+G-CSF treatment compared to WT mice. However, CCR5 knockout only partially blocks the SCF+G-CSF-induced adhesion of bone marrow-derived cells to endothelial cells. There might be other underlying mechanisms by which SCF+G-CSF treatment enhances the adhesion of bone marrow-derived blood cells to cerebral endothelial cells. In addition to the interaction of CCR5 and its ligands, other ligand-receptor interactions, such as the EphrinB2/EphA4, may also participate in SCF+G-CSF-enhanced monocyte adhesion to endothelial cells [60]. It is worth noting that in our in vitro experiments, blocking CCR5 fails to reduce monocyte-endothelial cell adhesion triggered by TNF-α or LPS (inflammatory mediators), indicating that inflammation-related monocyte-endothelial cell adhesion does not require CCR5, which differs from SCF+G-CSF-enhanced monocyte-endothelial cell adhesion.

Interestingly, in our in vivo study, bone marrow-derived Iba1^+^ cells were observed surrounding, but not within, the lumen of brain blood vessels, indicating that bone marrow-derived monocytes express Iba1 or differentiate into macrophages only after transmigrating from the blood vessels into the brain.

A limitation of our in vivo study is the inability to specifically delete CCR5 in endothelial cells due to the lack of CCR5-floxed mice. It has been shown that CCR5 is expressed in bone marrow-derived cells, neurons, glial cells, endothelial cells, and vascular smooth muscle cells [61]. Although bone marrow transplantation can rule out the effects of CCR5 in bone marrow-derived cells, CCR5 knockout mice may not only delete CCR5 in endothelial cells but also remove CCR5 from neurons, glial cells, and vascular smooth muscle cells. Therefore, we cannot draw a definitive conclusion in our in vivo study that our findings are specifically related to the knockout of CCR5 in endothelial cells.

The mechanisms by which monocytes adhere to cerebral endothelial cells and transmigrate into the perivascular space could be under the coordinated control of a wide range of signaling pathways, many of which are only just beginning to be understood. The findings presented here demonstrate that SCF+G-CSF plays a pivotal role in monocyte-endothelium adhesion and transmigration and further substantiate that CCR5 is involved in mediating SCF+G-CSF-enhanced monocyte-endothelium adhesion and transmigration. These findings shed new light on the biological function of SCF+G-CSF in endothelial cells and suggest a regulatory role of SCF+G-CSF in replenishing perivascular macrophages from monocytes in the adult brain under physiological conditions.

## 4. Materials and Methods

### 4.1. Animals

All experiments were carried out in accordance with protocols approved by the Institutional Animal Care and Use Committee and in keeping with the National Institute of Health’s Guide for the Care and Use of Laboratory Animals.

For the in vitro study, Iba1-GFP transgenic mice with a C57BL/6 background, originally developed by Dr. Kohsaka [62], were used. The original breeding pairs were kindly provided by Dr. Kohsaka. C57BL/6 mice (Jackson Laboratory, Bar Harbor, ME, USA) served as WT controls. Male mice aged 8–10 weeks were used for experiments.

For the in vivo study, transgenic mice ubiquitously expressing enhanced GFP under the control of the human ubiquitin C promoter (UBC-GFP) with a C57BL/6 background, CCR5 knockout (CCR5^−/−^) mice (C57BL/6 genetic background), and C57BL/6 mice were purchased from Jackson Laboratory (Bar Harbor, ME, USA). Male mice were used for experiments.

Mice were housed under a 12-h light/dark cycle with ad libitum access to food and water. 

### 4.2. Culture of bEnd.3 Endothelial Cells

The murine brain endothelial cell line bEnd.3 (ATCC, CRL-2299) was cultured in DMEM medium (ATCC, Manassas, VA, USA) with 10% fetal bovine serum (FBS; Atlanta Biologicals, Atlanta, GA, USA), 100 U/mL penicillin (Invitrogen, Carlsbad, CA, USA), and 100 µg/mL streptomycin (Invitrogen) at 37 °C under a humidified atmosphere of 5% CO_2_. Confluent monolayers were passaged by adding 0.25% trypsin-EDTA (Cellgro, Manassas, VA, USA) into 24 well cell culture plates.

The bEnd.3 cells at passages 20 to 30 were used for adhesion assays or mRNA analysis when they formed confluent endothelial cell monolayers. 

### 4.3. Isolation and Culture of Brain Endothelial Cells

C57BL/6 mice were deeply anesthetized and transcardially perfused with 30 mL of phosphate-buffered saline (PBS) to remove blood cells. The forebrain was removed and suspended in RPMI-1640 medium (ThermoFisher Scientific, Liverpool, NY, USA). The suspension was digested with type I collagenase (1 mg/mL, Worthington, NJ, USA) and DNase I (50 µg/mL, Roche Diagnostics, Indianapolis, IN, USA) at 37 °C for 45 min in a shaker set at 180 revolutions per minute. Brain endothelial cells were isolated using a 37–70% Percoll (GE Healthcare, Chicago, IL, USA) density gradient centrifugation method as described previously [63]. The endothelial cells were obtained from the interface, washed twice with DMEM medium, and resuspended in DMEM medium containing 10% FBS for phenotyping.

### 4.4. Isolation of Iba-1 Positive Monocytes from Bone Marrow Cells

Iba1^+^ monocytes were obtained from the bone marrow of Iba1-GFP mice. Iba1-GFP mice were used for the isolation of Iba1^+^ monocytes in this study because Iba1 is expressed in blood monocytes [64,65]. The Iba1-GFP mice were anesthetized and euthanized by cervical dislocation. The femur and tibia bones were removed, cleaned of all connective tissue, and placed on ice in complete bone marrow medium (CBMM). CBMM consisted of DMEM medium supplemented with 10% horse serum (HS; Hyclone Laboratories, Logan, UT, USA). The ends of each femur and tibia bone were clipped to expose the marrow. Syringes with 21-gauge needles were used to flush out the bone marrow cells from one end of the bone until it turned completely white. The cells were suspended in CBMM and centrifuged at 1500 rpm for 5 min at 4 °C. The cells were then resuspended in red cell lysis buffer (eBioscience, San Diego, CA, USA) and incubated at room temperature for 5 min. The cells were filtered through a 70 µm nylon mesh strainer (BD, Franklin Lakes, NJ, USA) and then washed three times with CBMM. The Iba-1^+^ monocytes were further sorted by Fluorescence Activated Cell Sorting (FACS, BD FACSVantage/Diva, Franklin Lakes, NJ, USA). Post-sorting reanalysis showed >95% purity of the Iba-1-GFP^+^ population. 

### 4.5. Analysis of Cells by Flow Cytometry

Anti-mouse antibodies used for flow cytometry included: rat anti-CD31 (MEC 13.3, BD Pharmingen, San Diego, CA, USA), rat anti-CD45-APC (30F11, eBioscience, San Diego, CA, USA), rabbit anti-mouse ckit (sc-168, Santa Cruz Biotechnology, Dallas, TX, USA), rabbit anti-mouse GCSFR (sc-694, Santa Cruz Biotechnology, USA), rabbit IgG (sc-3888, Santa Cruz Biotechnology, USA), anti-CCR5-PE (HM-CCR5 (7A4), eBioscience, USA), anti-CD106-FITC (VCAM-1, 429, eBioscience, USA), and anti-CD54-FITC (ICAM-1, YN1/1.7.4, eBioscience, USA). The brain single cell suspension was centrifuged, and the supernatant was removed. The cell pellets were fixed in cold 4% paraformaldehyde (PFA) (Polysciences, Inc., Warrington, PA, USA) at room temperature for 20 min and washed with staining medium (PBS containing 0.1% NaN_3_ and 2% FCS). The cells were incubated with primary antibodies (combinations of anti-CD31 and anti-ckit, anti-CD31 and anti-GCSFR, anti-CD31 and rabbit IgG) for 1 h and washed twice with staining medium. The cells were then incubated with Cy2-anti-Rat (1:200, Jackson ImmunoResearch Laboratories, West Grove, PA, USA) and Dylight-549-conjugated anti-Rabbit (diluted 1:400, Jackson ImmunoResearch Laboratories) for 30 min. The cells were washed again and incubated with anti-CD45-APC for an additional 30 min. An extra cell wash was performed before detection by flow cytometry (FACSCalibur, BD science, Franklin Lakes, NJ, USA). For bEnd.3 cells, the cultured cells were rinsed with 0.25% trypsin-EDTA to get a single cell suspension and processed following the same staining procedures as stated above. Data were analyzed using FlowJo software 9.3.2 (TreeStar, Ashland, OR, USA). Brain endothelial cells were gated on the CD45^−^/CD31^+^ population and mean fluorescence intensity (MFI) was presented relative to appropriate isotype controls. All information about antibodies used for flow cytometry is provided in Table 1.

### 4.6. Adhesion Assay

The adhesion experiments were performed as previously described [66]. bEnd.3 monolayers were incubated with recombinant mouse SCF (PeproTech, Rocky Hill, NJ, USA) and/or recombinant human G-CSF (Amgen, Thousand Oaks, LA, USA) for 16–18 h at the indicated concentrations. TNF-α (100 ng/mL) (Life Technologies, Grand Island, NY, USA) and LPS (1 µg/mL) (Sigma, St. Louis, MO, USA) served as positive controls. The stimulated bEnd.3 cells were washed three times with pre-warmed 10% fetal calf serum (FCS) in DMEM. Iba1^+^ monocytes (500,000 cells in a volume of 200 µL per well) were then added on the top of bEnd.3 cells. In some experiments, stimulated bEnd.3 cells were incubated with anti-mouse-CCR5 (10 µg/mL) (HM-CCR5 (7A4), eBioscience, USA) or Armenian Hamster IgG isotype control antibody (eBio299Arm, eBioscience, USA) for 30 min at 37 °C and washed before the addition of Iba1^+^ monocytes to the bEnd.3 cells. The cell culture plates were incubated at room temperature on a horizontal shaker (70 rpm) for 20 min. The plates were then washed twice to remove unbound or loosely bound cells and fixed in 4% PFA in PBS for 20 min. Binding was determined by counting the adherent GFP^+^ cells in four randomly chosen microscope fields (X20) in each well using a Nikon Eclipse (TE2000) inverted fluorescence microscope (ECLIPSE TE2000-s, Nikon, Tokyo, Japan). The cells from four fields were averaged for data analysis. Data collection was blinded to the experimental treatments.

### 4.7. Real-Time Quantitative Polymerase Chain Reaction

Total RNA was extracted from cultured cells using an RNeasy Plus Mini Kit (Qiagen, Hilden, Germany). First strand complementary DNA (cDNA) was prepared from an RNA template (1 µg) using the High-Capacity RNA-to-cDNA kit (Applied Biosystems, Waltham, MA, USA). Reverse transcription was performed at 37 °C for 60 min, followed by 95 °C for 5 min, according to the manufacturer’s instructions. Using the SsoAdvanced SYBR Green Supermix (Bio-Rad, Hercules, CA, USA), real-time quantitative polymerase chain reaction (RT-qPCR) amplification was performed by enzyme activation at 95 °C for 30 s, denaturation at 95 °C for 5 s, and annealing and extension at 58 °C for 1 min. Forty cycles of this process were repeated, followed by melting curve analysis for each reaction to confirm single amplified products between 65–95 °C in 0.2 °C increments on the Bio-Rad CFX96 system (Bio-Rad, USA). The relative quantification of mRNA of target genes across several time points was determined by calculating the ratio of CT values divided by the CT values of glyceraldehyde-3-phosphate dehydrogenase (GAPDH) from the corresponding time points. All primers were ordered from Integrated DNA Technologies. Primer sequences are listed in Table 2.

### 4.8. Experimental Design of In Vivo Study

Mice were divided into four experimental groups at 3 months of age: a WT-vehicle control group (n = 5), a WT-SCF+G-CSF group (n = 5), a CCR5^−/−^-vehicle control group (n = 5), and a CCR5^−/−^-SCF+G-CSF group (n = 5). As described in our earlier study [20], after receiving a lethal dose of whole-body irradiation (9 gray), bone marrow transplantation (BMT) was performed within 24 h. One month after BMT, recombinant mouse SCF (200 μg/kg/day, diluted in saline) (PeproTech, Rocky Hill, NJ, USA) and recombinant human G-CSF (50 μg/kg/day, diluted in 5% dextrose) (Amgen, Thousand Oaks, CA, USA) or an equal volume of vehicle solution (50% saline and 50% dextrose), were subcutaneously injected for 5 consecutive days. Mice were sacrificed 6–8 h after the final injection. 

### 4.9. Bone Marrow Transplantation

UBC-GFP mice were deeply anesthetized. The femur and tibia bones were removed, cleaned of all connective tissue, and placed on ice in Hanks’ Balanced Salt Solution (HBSS). As described earlier, the ends of each tibia and femur were clipped to expose the marrow. The bone marrow was flushed out using ice-cold HBSS with a syringe and a 21-gauge needle. The bone marrow cells were filtered through a 70 µm nylon mesh strainer (BD, USA), centrifuged at 300× *g* for 10 min at 4 °C, and resuspended in ice-cold HBSS into a single cell suspension. Cells were transplanted into irradiated CCR5^−/−^ or WT mice within 24 h after irradiation (9 gray) by tail vein injection (1 × 10^7^ bone marrow cells in 0.7 mL HBSS per mouse).

### 4.10. Immunofluorescence Staining

Mice were deeply anesthetized and perfused through the left cardiac ventricle with cold PBS, followed by 10% phosphate buffered formalin (Fisher scientific, Waltham, MA, USA). Brains were removed and immersed in the same fixative overnight at 4 °C, then cryoprotected with 30% sucrose in PBS at 4 °C for 2 days. Coronal brain sections, 30 µm thick, were cut with a cryostat (Leica Biosystems, Wetzlar, Germany). A free-floating technique was used for immunofluorescence staining. Briefly, brain sections (3–4 adjacent sections/brain) (1.10 mm posterior to bregma) were selected for immunofluorescence staining. The sections were rinsed with PBS and incubated in a solution of PBS-diluted 10% normal goat serum or normal donkey serum (Jackson ImmunoResearch Laboratories, West Grove, PA, USA), containing 1% bovine serum albumin (BSA) (Sigma-Aldrich, St. Louis, MO, USA) and 0.3% Triton X-100 (Sigma-Aldrich, USA) for 1 h at room temperature to block non-specific binding. The sections were then incubated with the primary antibodies including rabbit anti-Ckit (1:100, Santa Cruz Biotechnology, Dallas, TX, USA), rabbit anti-GCSFR (1:100, Santa Cruz Biotechnology, USA), rat anti-CD31 (1:200, MEC 13.3, BD Pharmingen, San Diego, CA, USA), rabbit anti-CD31 (1:50, Abcam, Cambridge, UK), rabbit anti-Iba1 (1:400, Wako, Osaka, Japan), and goat anti-GFP (FITC-conjugated) (1:300, Abcam, UK), overnight at 4 °C. The primary antibodies were diluted in PBS solution with 1% BSA and 0.3% Triton X-100. In negative control brain sections, the primary antibodies were omitted. The following day, the sections were washed with PBS and incubated with the secondary antibodies diluted in PBS solution with 1% BSA and 0.3% Triton X-100 at room temperature for 2 h in the dark. The secondary antibodies used in this study were Cy2-conjugated goat anti-rat (1:200, Jackson ImmunoResearch Laboratory, USA), Dylight-549-conjugated goat anti-rabbit (1:400, Jackson ImmunoResearch Laboratory, USA), Alexa-Fluor 594-conjugated donkey anti-rabbit (1:500, Life Technologies, Carlsbad, CA, USA), and Alexa-Fluor 488-conjugated donkey anti-goat (1:500, Life Technologies, USA). For triple immunofluorescence staining, the primary antibodies used were rat anti-Ki67 (1:100, Invitrogen, Carlsbad, CA, USA), rabbit anti-Iba1 (1:500, Wako, Osaka, Japan), and goat anti-GFP (1:500, Novus Biologicals, Centennial, CO, USA), and the secondary antibodies used were Alexa-Fluor 647-conjugated donkey anti-rat (1:400, Life Technologies, Carlsbad, CA, USA), Alexa-Fluor 594-conjugated donkey anti-rabbit (1:400, Life Technologies, USA), and Alexa-Fluor 488-conjugated donkey anti-goat (1:400, Life Technologies, USA). After incubation with the secondary antibodies, the sections were rinsed with PBS, mounted on Superfrost Plus slides (ThermoFisher Scientific, Liverpool, NY, USA), and coverslipped with Vectashield Antifade Mounting Medium (Vector Laboratories, Newark, CA, USA). Z-stack images of 15 layers with an interval of 1.0 μm in the cortex were taken with an LSM 780 confocal microscope (Zeiss, Oberkochen, Germany) or a Leica SP8 confocal microscope (Leica Microsystems, Wetzlar, Germany) for the triple immunofluorescence staining. Bone marrow-derived cells adhering to the endothelial cells were determined by the co-localization of GFP^+^ cells and CD31^+^ cells under the orthogonal view using ImageJ software 9.3.2. Monocytes/macrophages surrounding blood vessels were defined as GFP^+^/Iba1^+^ monocytes/macrophages within 10 µm of CD31^+^ blood vessels. The number of GFP^+^ cells adhering to the CD31^+^ endothelial cells, and the number of GFP^+^/Iba1^+^ monocytes/macrophages surrounding the blood vessels were quantified. GFP^+^/Iba1^+^ Ki67^+^ cells were also quantified using ImageJ software.

In the in vitro experiment, bEnd.3 cells were fixed in 4% cold PFA (Polysciences, Inc., PA, USA), and immunofluorescence staining was performed following the same procedures as stated above.

### 4.11. Statistical Analysis

Data analysis was performed in a blinded manner. Two-tailed *t*-tests were used to determine significant differences between two groups. One-way or two-way ANOVA followed by post hoc Tukey’s test was used for comparisons among three or more groups. For all tests, probability values < 0.05 were considered statistically significant. Data are presented as mean ± SEM.

## Figures and Tables

**Table 1:** Antibodies for flow cytometry.

Antibodies	Clone Information	Source	Dilution
Rat anti-CD31	MEC 13.3	BD Pharmingen, San Diego, CA, USA	1:100
Rat anti-CD45	30F11	eBioscience, San Diego, CA, USA	1:1000
Rabbit anti-mouse ckit	sc-168	Santa Cruz Biotechnology, Dallas, TX, USA	1:200
Rabbit anti-mouse GCSFR	sc-694	Santa Cruz Biotechnology, USA	1:200
Rabbit IgG	sc-3888	Santa Cruz Biotechnology, USA	1:200
Anti-mouse CCR5 (CD195)	HM-CCR5 (7A4)	eBioscience, USA	1:200
Hamster IgG Isotype Control	eBio299Arm	eBioscience, USA	1:200
Anti-mouse CD106 (VCAM-1)	429	eBioscience, USA	1:200
Anti-mouse CD54 (ICAM-1)	YN1/1.7.4	eBioscience, USA	1:200

**Table 2:** Primers for PCR.

Target Gene	Sequences	Length (bp)
GAPDH	sense 5′-CCA TCA CCA TCT TCC AGG AG-3′ antisense 5′-GTG GTT CAC ACC CAT CAC AA-3′	194
ICAM-1	sense 5′-GCC TCC GGA CTT TCG ATC TT-3′ antisense 5′-GTA GAC TGT TAA GGT CCT CTG CGT-3′	304
E-selectin	sense 5′-GAT TGG ACA CTC AAT GGA TC-3′ antisense 5′-CCT AGA CGT TGT AAG AAG GC-3′	271
P-selectin	sense 5′-CTA TAC CTG CTC CTG CTA CCC AGG C-3′ antisense 5′-TTC ACT CCA CTG ACC AGA GCC AGT G-3′	403
VCAM-1	sense 5′-TGC CGA GCT AAA TTA CAC ATT G-3′ antisense, 5′-CCT TGT GGA GGG ATG TAC AGA	124
CCR5	sense 5′-CAA GAC AAT CCT GAT CGT GCA A-3′ antisense 5′-TCC TAC TCC CAA GCT GCA TAG AA-3′	128
CCL1	sense 5′-CCG TGT GGA TAC AGG ATG TTG-3′ antisense 5′-TCAG GAC AGG AGG AGC CC-3′	133
CCL3	sense 5′-ACC ACT GCC CTT GCT GTT C-3′ antisense 5′-TCT GCC GGT TTC TCT TAG TCA G-3′	202
CCL4	sense 5′-CTC TCC TCT TGC TCG TGG C-3′ antisense 5′-GTA CTC AGT GAC CCA GGG CTC-3′	229
CCL5	sense 5′-GCT GCC CTCA CCA TCA TCC-3′ anti-sense 5′-GTA TTC TTG AAC CCA CTT CTT CTC TG-3′	237
CX3CL1	sense 5′-CCG CGT TCT TCC ATT TGT GT-3′ antisense 5′-GCA CAT GAT TTC GCA TTT CG-3′	71

## Data Availability

The datasets generated during the current study are available from the corresponding author on reasonable request.

## Supplementary Materials

The following supporting information can be downloaded at: https://www.mdpi.com/article/10.3390/ijms25168898/s1.
