# Supplementary material for: Hematopoietic Growth Factors Regulate the Entry of Monocytes into the Adult Brain via Chemokine Receptor CCR5
Source: Int J Mol Sci. 2024 Aug 15;25(16):8898. doi: 10.3390/ijms25168898 (PMC11354986; doi:10.3390/ijms25168898)
Supplement: Supplementary file 1 [file ijms-25-08898-s001.zip › ijms-3036698-Supplementary.pdf]

Supplementary Figure Legends

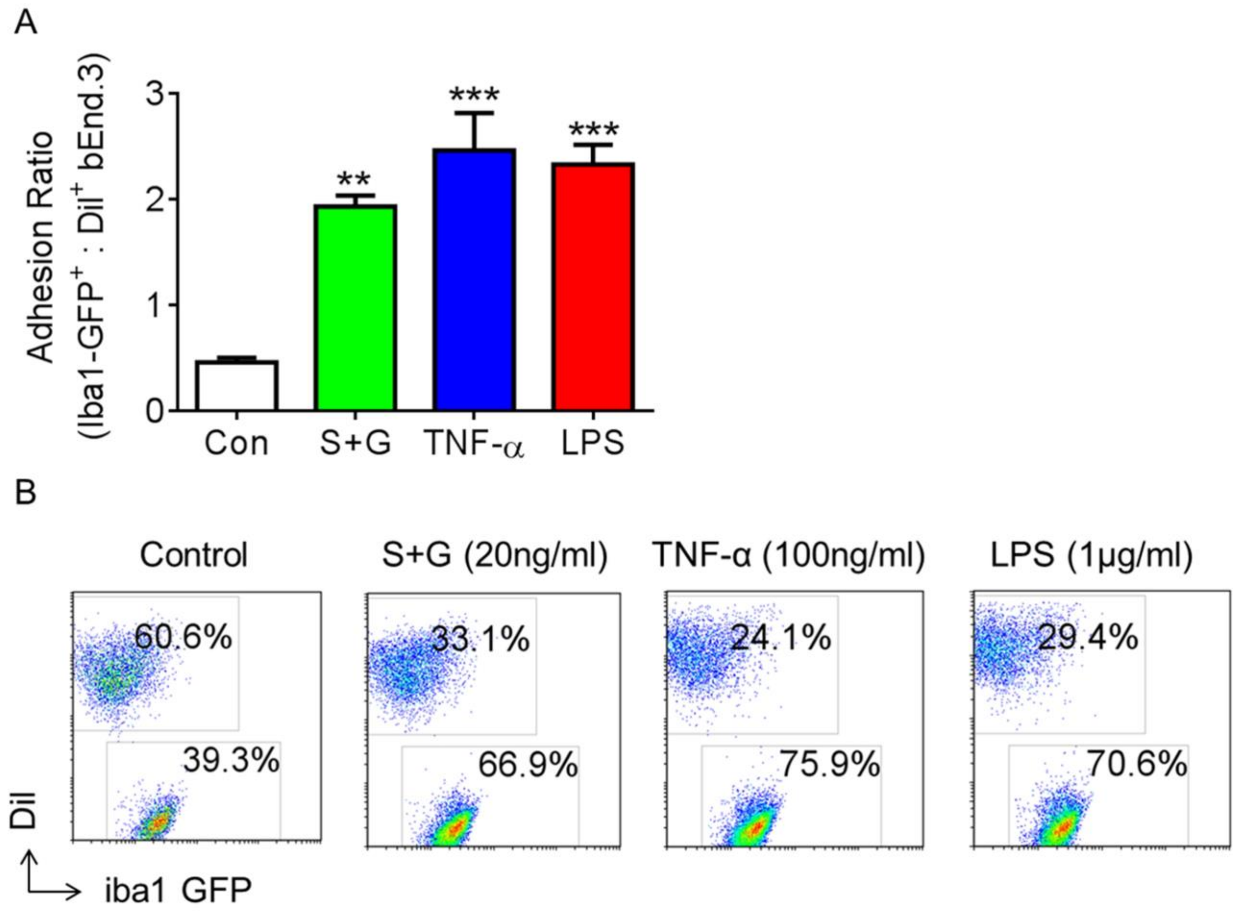

Supplementary Figure S1. SCF in combination with G-CSF enhances Iba-1^{+} monocyte adhesion to endothelial cells. (**A**) Quantification data. Mouse brain-derived endothelial cells (i.e. bEnd.3 cells) were incubated with medium alone (con: control), SCF+G-CSF (20ng/ml), TNF-*α* (100ng/ml), and LPS (1μg/ml) for 16-18 hours. After washing, Iba-1-GFP^{+} monocytes were added to the bEnd.3 cells. The monocyte-endothelial cell adhesion assay was performed using flow cytometry. Mean ± SEM. Repeated by 3 independent experiments. $^{**}p < 0.01$, $^{***}p < 0.001$ *vs.* the medium control. One-way ANOVA followed by *post-hoc* Tukey's test. (**B**) Representative dot plots of flow

cytometry showing adhesion of Iba-1-GFP⁺ monocytes to bEnd.3 cells that were pre-treated with medium alone, SCF+G-CSF (20ng/ml), TNF-α (100ng/ml), and LPS (1μg/ml) for 16-18 hours.

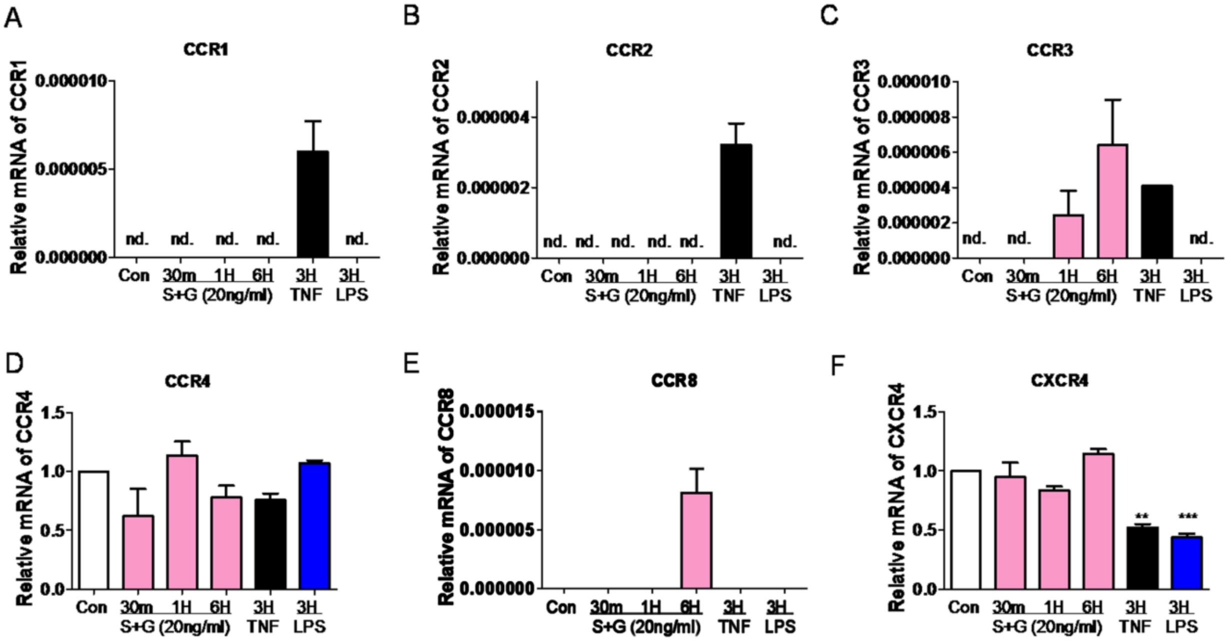

Supplementary Figure S2. The mRNA expressions of other chemokine receptors on bEnd.3 cells are not changed by SCF+G-CSF treatment. (**A-F**) Quantitative real-time PCR data. The mRNA expressions of CCR1 (**A**), CCR2 (**B**), CCR3 (**C**), CCR4 (**D**), CCR8 (**E**), and CXCR4 (**F**) in bEnd.3 cells that were cultured for different time periods (30min to 6 hours) in the presence of medium alone (con: control), SCF+G-CSF (20ng/ml), TNF-α (100ng/ml), and LPS (1μg/ml). Mean ± SEM. Repeated by 3 independent experiments. **$p < 0.01$, ***$P < 0.001$ *vs.* medium control. One-way ANOVA followed by *post-hoc* Tukey's test. nd: not detected.

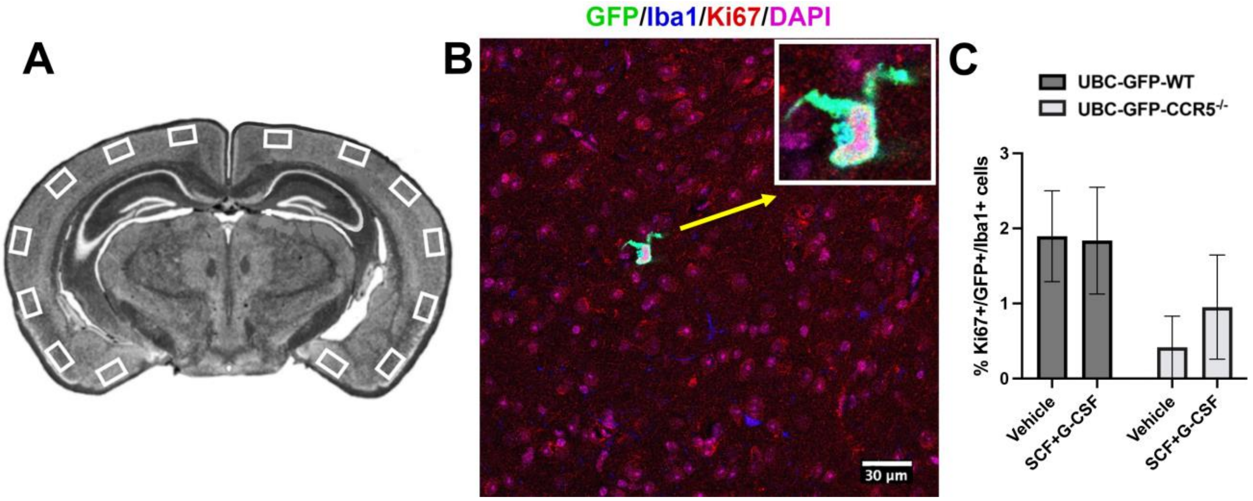

Supplementary Figure S3. Ki67/Iba1/GFP positive cells in the cortex of adult brain. (**A**) A diagram indicates the selected regions in the cortex for confocal imaging. (**B**) A representative projection view of z-stack confocal images. Boxed image is the enlarged image showing a GFP/Iba1/Ki67/DAPI positive cell (i.e., a bone marrow-derived macrophage with proliferation) in the cortex. (**C**) Quantification data show the percentage of Ki67/Iba1/GFP positive cells in all Iba1/GFP positive cells. Mean ± SEM. N=5. Two-way ANOVA followed by *post-hoc* Tukey's test.
